# Cerebrospinal fluid is drained primarily via the spinal canal and olfactory route in young and aged spontaneously hypertensive rats

**DOI:** 10.1186/2045-8118-11-12

**Published:** 2014-06-06

**Authors:** Lucy A Murtha, Qing Yang, Mark W Parsons, Christopher R Levi, Daniel J Beard, Neil J Spratt, Damian D McLeod

**Affiliations:** 1 University of Newcastle and Hunter Medical Research Institute, University of Newcastle: School of Biomedical Sciences & Pharmacy, Medical Sciences Building, Callaghan, NSW 2308, Australia; 2 Apollo Medical Imaging Technology Pty Ltd, Suite 611, 365 Little Collins Street, Melbourne, Vic 3000, Australia; 3 Hunter New England Local Health District: Department of Neurology, John Hunter Hospital, Locked Bag 1, Hunter Region M.C, NSW 2310, Australia

**Keywords:** Computed tomography, Cerebrospinal fluid dynamics, Contrast, Spontaneously hypertensive rat, Intracranial pressure (ICP), Age, CSF, SHR

## Abstract

**Background:**

Many aspects of CSF dynamics are poorly understood due to the difficulties involved in quantification and visualization. In particular, there is debate surrounding the route of CSF drainage. Our aim was to quantify CSF flow, volume, and drainage route dynamics *in vivo* in young and aged spontaneously hypertensive rats (SHR) using a novel contrast-enhanced computed tomography (CT) method.

**Methods:**

ICP was recorded in young (2–5 months) and aged (16 months) SHR. Contrast was administered into the lateral ventricles bilaterally and sequential CT imaging was used to visualize the entire intracranial CSF system and CSF drainage routes. A customized contrast decay software module was used to quantify CSF flow at multiple locations.

**Results:**

ICP was significantly higher in aged rats than in young rats (11.52 ± 2.36 mmHg, versus 7.04 ± 2.89 mmHg, *p* = 0.03). Contrast was observed throughout the entire intracranial CSF system and was seen to enter the spinal canal and cross the cribriform plate into the olfactory mucosa within 9.1 ± 6.1 and 22.2 ± 7.1 minutes, respectively. No contrast was observed adjacent to the sagittal sinus. There were no significant differences between young and aged rats in either contrast distribution times or CSF flow rates. Mean flow rates (combined young and aged) were 3.0 ± 1.5 μL/min at the cerebral aqueduct; 3.5 ± 1.4 μL/min at the 3rd ventricle; and 2.8 ± 0.9 μL/min at the 4th ventricle. Intracranial CSF volumes (and as percentage total brain volume) were 204 ± 97 μL (8.8 ± 4.3%) in the young and 275 ± 35 μL (10.8 ± 1.9%) in the aged animals (NS).

**Conclusions:**

We have demonstrated a contrast-enhanced CT technique for measuring and visualising CSF dynamics *in vivo.* These results indicate substantial drainage of CSF via spinal and olfactory routes, but there was little evidence of drainage via sagittal sinus arachnoid granulations in either young or aged animals. The data suggests that spinal and olfactory routes are the primary routes of CSF drainage and that sagittal sinus arachnoid granulations play a minor role, even in aged rats with higher ICP.

## Background

Cerebrospinal fluid (CSF) dynamics are thought to be altered in many pathological conditions including hydrocephalus [1,2], idiopathic intracranial hypertension [3,4], intracerebral haemorrhage [5], subarachnoid haemorrhage [6,7], large hemispheric stroke [8,9], traumatic brain injury [10], and in the aging brain [11,12]. However, relatively little is known about the exact mechanisms of changes in CSF dynamics and drainage in these conditions due to difficulties in quantification.

The role and location of CSF drainage has been studied in both animals and humans. Traditional interpretation has been that most CSF drains into the venous sinuses via arachnoid granulations [13-15]. The primary site of CSF reabsorption, however, has become a contentious issue over the last decade. The importance of the olfactory perineural pathways and the cervical lymphatics [16-19] in reabsorbing CSF has been studied in several species, including humans. Postmortem lymphatic vascular casting [20-22] and radioactive albumin clearance methods [17,23-25] have demonstrated the major contribution of these pathways in CSF drainage. Furthermore, physiological studies by Johnston’s group, suggested that the arachnoid granulations may only come into play when intracranial pressure is elevated and that lymphatic drainage routes may play a major role [26,27], especially in neonates where arachnoid granulations are sparse [28]. Both human and animal studies indicate that the spinal route, either via spinal arachnoid granulations or via lymphatics around spinal nerve root dural sheaths may also be important [29-35]. These lymphatic routes may have important immunological significance [19].

To investigate CSF dynamics and drainage within the entire rat brain we developed a novel contrast-enhanced computed tomography (CT) technique to image the rat CSF system in three-dimensions. One unique feature of this technique is that the dynamic nature of CSF drainage can be observed *in vivo*. We chose to compare young and aged spontaneously hypertensive rats (SHR) because their cerebral ventricular volume is thought to increase with age [36,37], which, in addition to the hypertension, may affect CSF dynamics. In addition, this effect may also influence physiological variables such as intracranial pressure, for example, elevated ICP is seen in some hydrocephalus patients [1,2]. Using our novel contrast-enhanced CT method, we hypothesized that the aged SHRs would have altered CSF dynamics and drainage and a higher baseline ICP when compared to the young SHRs.

## Methods

All animal experiments were conducted in accordance with the Australian Code of Practice for the Care and Use of Animals for Scientific Purposes and were approved by The University of Newcastle Animal Care and Ethics Committee. Experiments were performed on two cohorts of male spontaneously hypertensive rats (SHR) (Animal Resources Centre, Western Australia). One group weighed 200-360 g, aged 2–5 months (n = 5); the other group weighed 360-400 g, aged 16 months (n = 5). Due to ethical constraints, we calculated the sample size required to detect a difference in baseline ICP between two different rat strains (Wistar and Long Evans; data previously published [38]). Using an alpha of 0.05 and Power of 0.80, a sample size of 5 animals per group was required to detect a 4.2 mmHg difference between baseline ICP in the two rat strains.

### Surgery and physiological monitoring

Animals were anaesthetised with isoflurane (5% induction, 2% maintenance) in 50:50%, N_2_:O_2_ via a facemask. It has previously been reported in dogs that isoflurane causes no significant change in the rate of CSF production [39]. Respiratory rate was regularly monitored and core temperature was maintained at 37°C via a thermocouple rectal probe and warming plate for the duration of surgical anaesthesia. Incision sites were shaved, cleaned and injected subcutaneously with 2 mg/kg 0.05% Bupivacaine (Pfizer, Australia). To measure arterial blood pressure under anaesthetic, a fibreoptic microcatheter (SAMBA Sensors, Sweden) was briefly inserted into the saphenous branch of the femoral artery, and a steady state baseline arterial blood pressure trace was recorded.

Intracranial pressure (ICP) was recorded prior to scanning using a SAMBA microcatheter as previously described [40] with minor changes. Briefly, animals were placed in the ear bars of a custom-built CT-compatible stereotaxic frame. Hollow poly-ether-ether-ketone (PEEK) screws (Solid Spot LLC, Santa Clara, CA, USA) of 2 mm in diameter × 5 mm in length were inserted bilaterally 0.3 mm caudal and 1.5 mm lateral to Bregma. The ICP probe was inserted into the right screw and an airtight seal made by surrounding both screws in a biocompatiable caulking material (Silagum, DMG Dental, Hamburg, Germany). The probe was removed prior to scanning.

### Computed tomography (CT) imaging

**Figure 1:**
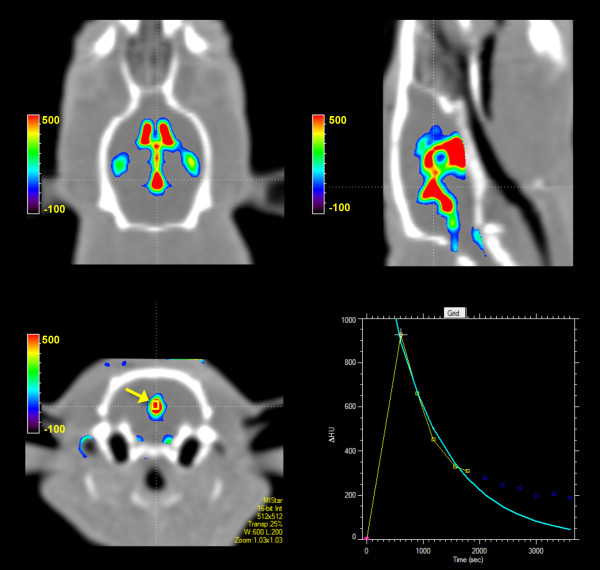
**Calculating cerebrospinal fluid flow through the cerebral aqueduct of the rat.** Radio-opaque contrast (20 μl) was simultaneously injected into each lateral ventricle at 2 μl/min for 10 min while plain CT images (0.6 mm slice thickness) were taken over 60 minutes. A small region-of-interest (ROI) (yellow box, arrow) was positioned within the centre of the aqueduct and the decay rate used to generate flow maps. An additional movie file shows this in more detail [see Additional file 1].

Following baseline arterial pressure and ICP measurement, animals remained on the same base plate and stereotaxic frame, which was positioned on the CT scanner table. All imaging was performed using a 64-slice clinical CT scanner (Siemens, Erlangen, Germany) [41,42]. The CSF imaging protocol was developed specifically for the current study. Each CT-CSF imaging sequence used 0.6 mm slice thickness with coronal plane image acquisition, and a total of 90 slices captured from the rat nose to the cervical vertebrae (C2). Two 1 ml syringes and two PE-10 intraventricular catheters were then preloaded with a 1:4 dilution of Ultravist 300 mg/mL (Bayer HealthCare Pharmaceuticals Inc.) in 0.9% saline. This dilution was chosen to reduce the viscosity and make it closer to that of CSF. Care was taken to ensure that no air bubbles were present within the catheters. The catheter tips were inserted 3.5 mm below the skull into each lateral ventricle via the bilateral screws used for ICP monitoring, and a head CT scan performed (baseline scan) to ensure that catheter tips were positioned within each lateral ventricle. Using an automated syringe driver (Harvard Apparatus, Pump 11 Elite, MA, USA), contrast was injected into each ventricle at 2 μL/min for 10 minutes, with a CT scan performed every minute from the start of infusion. The infusion rate of 2 μL/min was chosen to reduce the possibility of the infusion affecting the CSF production (rodent CSF production rate previously reported as 2.66-2.84 μL/min [43]). Following the cessation of infusion, serial CT scans were performed every 5 minutes for 60 minutes (Figure 1, an additional movie file shows this in more detail [see Additional file 1]).

### Processing CT images

For each animal, serial CT images were loaded into MiStar software (Apollo Medical Imaging Technology Pty Ltd, Melbourne, Australia). Motion correction was performed, and the resulting images were subtracted from the baseline non-contrast image.

#### 3D reconstruction of CT images: rat CSF system

A representative 3D reconstruction of the rat CSF system was created by generating a Maximum Intensity Projection (MIP).The MIP was loaded into the MiStar fusion 3D render module. Thresholds were applied to the 3D render to highlight the CSF system.

#### Visual inspection of contrast time-course throughout CSF system

The time taken for injected contrast to reach specific anatomical landmarks within the CSF system was quantified in each animal. The anatomical landmarks included the cerebral aqueduct, 3rd ventricle, 4th ventricle, spinal canal, basal cisterns and cribriform plate. The contrast window range was set to 0–250 Hounsfield Units (HU) for each animal sequence to prevent the false detection of signal noise at each image time-point. The time taken for the contrast to reach each landmark was calculated by analysing each sequential image.

#### CSF flow rate calculations

Contrast-enhanced CT images were processed using the MiStar software decay module specifically written for rat imaging. CSF flow rate measured at the cerebral aqueduct was used to estimate CSF production. Flow rates were also calculated elsewhere within the CSF system. A small region-of-interest (ROI) with 6 voxels was positioned within the centre of the aqueduct on the 10 minute time-point scan (post-infusion). The total change in Hounsfield Units for voxels within the ROI was plotted over time, and an exponential decay curve was fitted to the first five decay data points. The exponential decay rate constant is directly proportional to flow within the ROI. This principle was applied to each voxel to generate the Decay Rate Map, measured in ml/min/100 L (which was then converted to μl/min/100 ml). The ROI was then co-registered with the Decay Rate Map to obtain the flow value. Flow was converted into μl/min by multiplication with volume (slice thickness × area of ROI). The process was repeated (with the same fixed ROI) at the 3rd ventricle and 4th ventricle of each animal.

#### Brain and intracranial CSF volume calculation

Twenty five 0.6 mm coronal CT slices were analysed slice-by-slice to calculate brain and CSF volume. On each slice, a threshold of 100 HU was set and ROI software tools were used to define the area of total brain tissue (using baseline scans) or contrast-enhanced CSF within the cranium. On each slice, the volume was calculated as slice thickness × area of ROI. The combined sum of the ROI volumes was calculated.

### Statistics

Statistical tests were performed using GraphPad Prism Version 6 for Windows (GraphPad Software, USA). Two-tailed Student’s t-test was used to compare differences in CSF production rate, and CSF volumes between young and aged groups. Significance was accepted at the *p* < 0.05 level. T-tests are also reported for physiological variables for illustrative purposes, with p-values uncorrected for use of multiple comparisons. Unless otherwise stated, data is expressed as mean ± standard deviation (±SD).

## Results

### Physiological variables

Arterial blood pressure (systolic/diastolic) was measured in 3 young animals and 5 aged animals. Mean arterial blood pressure was lower in the young versus the aged animals (94 ± 32 mmHg vs. 160 ± 22 mmHg, *p* = 0.01). Intracranial pressure (ICP) was measured in all animals and traces revealed consistent pulse and respiratory waveforms. A significantly higher ICP was found in aged rats, 11.52 ± 2.36 mmHg, versus 7.04 ± 2.89 mmHg in the younger animals, *p* = 0.03. Cerebral perfusion pressure (mean arterial blood pressure - intracranial pressure) was significantly lower in the young versus the aged animals (86 ± 32 mmHg vs. 148 ± 20 mmHg, *p* = 0.02).

### Cerebrospinal fluid drainage pathways and time course

**Figure 2:**
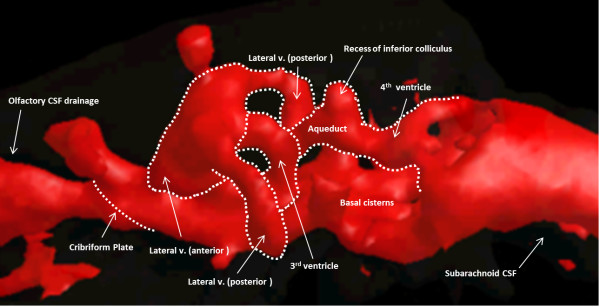
**Cerebrospinal fluid (CSF) system in a rat imaged with contrast-enhanced computed tomography (CT).** Images show a 3D render reconstruction of CT images after 20 μL of radio-opaque contrast was injected into each lateral ventricle via cannulae guided through hollow screws inserted into the skull. Anatomical landmarks of the CSF system in the rat are based on a stereotaxic rat atlas [61]. Basal cisterns are not depicted in the rat atlas, but their location at the base of the brain is indicated by presence of contrast enhanced CSF in this location. An additional movie file shows this in more detail [see Additional file 2].

**Figure 3:**
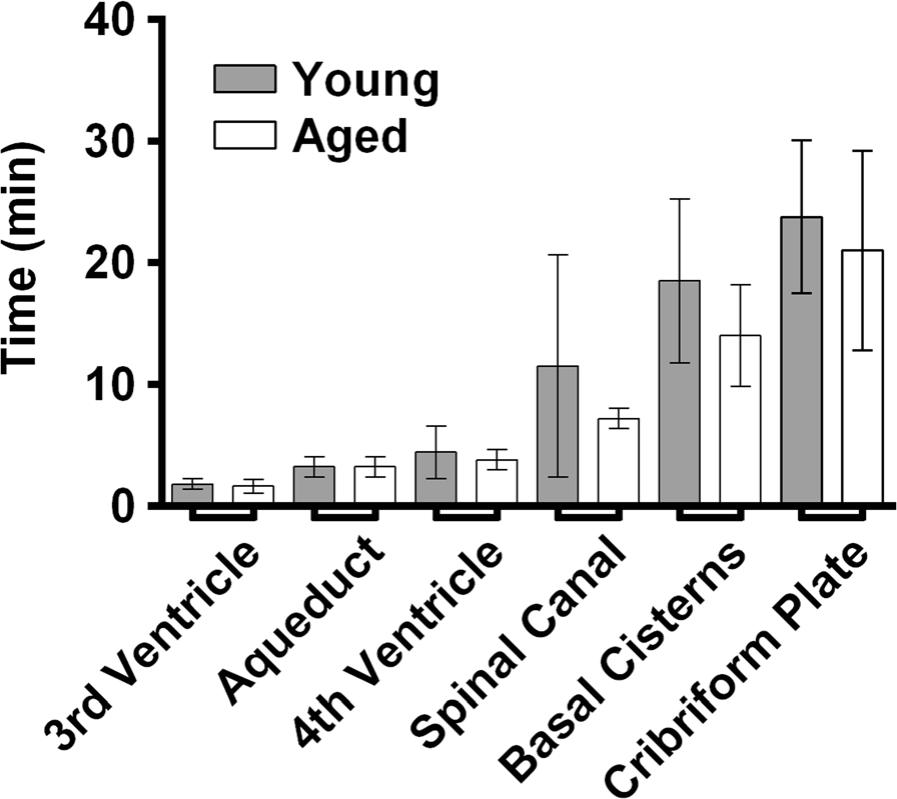
**Time taken for contrast-enhanced cerebrospinal fluid to reach major anatomical landmarks within the rat CSF system.** Young (2–5 months); Aged (16 months). Mean ± SD.

CSF was observed to drain into the spinal canal (subarachnoid space) and via the olfactory pathway (through the cribriform plate), the optic nerves, and the cervical lymph nodes in all animals (Figure 2, an additional movie file shows this in more detail [see Additional file 2]). Contrast-enhanced CSF was observed at the cerebral aqueduct 3.2 ± 0.8 minutes post-injection in both cohorts. It was first observed to enter the 3rd ventricle 1.8 ± 0.4 minutes and 1.6 ± 0.5 minutes post-injection in the young and aged respectively, the 4th ventricle at 4.4 ± 2.2 minutes and 3.8 ± 0.8 minutes post-injection, the spinal canal at 11.5 ± 9.1 minutes and 7.2 ± 0.8 minutes post-injection, and the basal cisterns at 18.5 ± 6.8 minutes and 14.0 ± 4.2 minutes post-injection in the young and the aged respectively (Figure 3). Contrast could be observed leaving the cranial vault at the cribriform plate 23.8 ± 6.3 minutes post-injection in the young and 21 ± 8.2 minutes in the aged (Figure 3). No contrast-enhanced CSF was observed at the sagittal sinus [Additional file 1].

### Cerebrospinal fluid production and flow rates and total intracranial CSF volume

**Figure 4:**
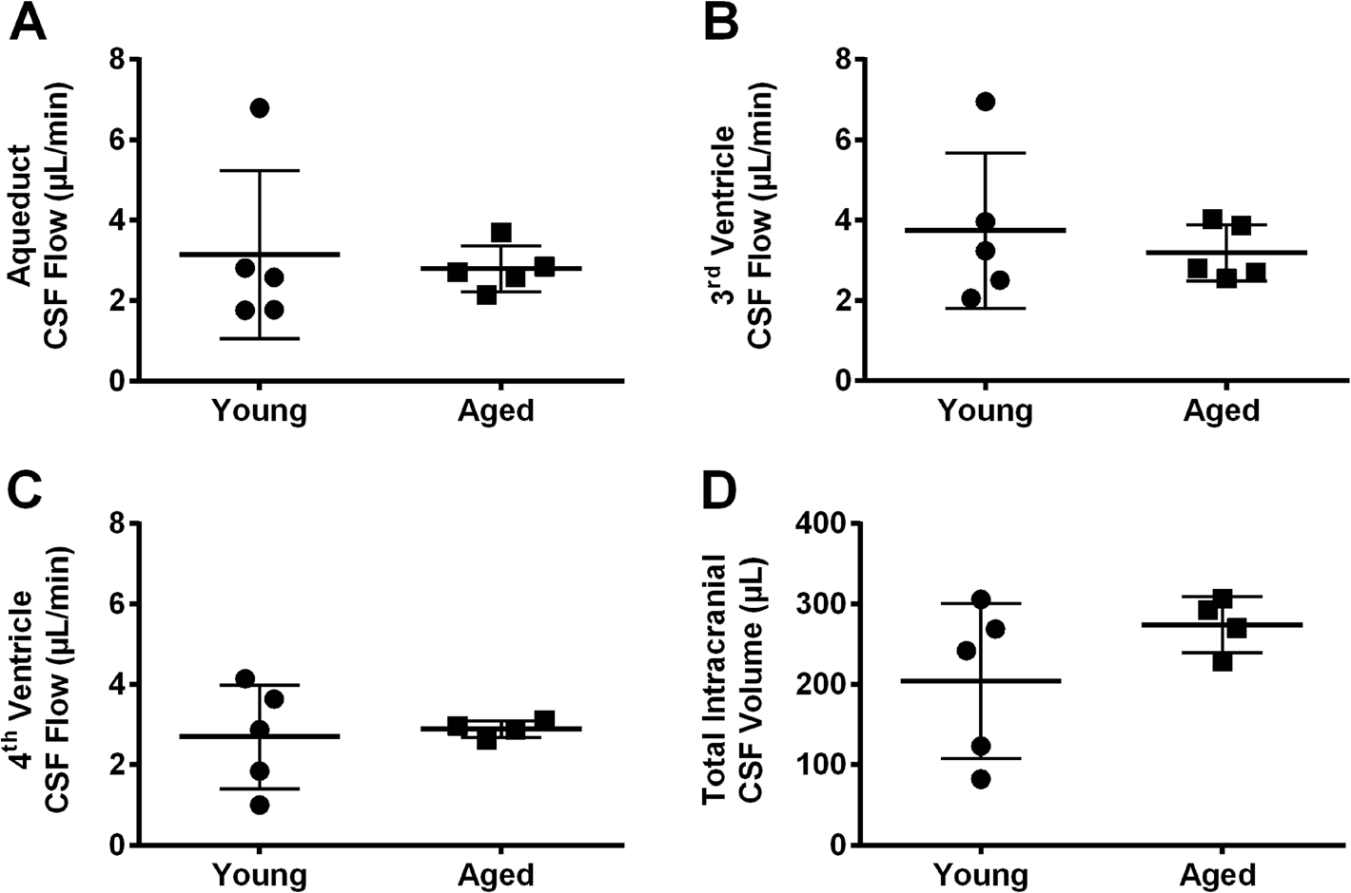
**Cerebrospinal fluid (CSF) dynamics in the rat. (A)** Flow rate through the cerebral aqueduct; **(B)** Flow rate through the 3rd ventricle; **(C)** Flow rate through the 4th ventricle; **(D)** Total intracranial CSF volume, in young (2–5 months) versus aged (16 months) spontaneously hypertensive rats. Data presented as individual data points with mean ± SD.

CSF flow was able to be quantified at multiple sites within the ventricular system, but flow quantification in the extraventricular locations was not possible due to respiratory motion artifact (spinal canal) and contrast dilution, resulting in reduced signal-to-noise ratio and partial volume averaging error caused by the extensive surface area of each drainage route. CSF production rates, as measured by CSF flow rate at the cerebral aqueduct, were 3.14 ± 2.1 μL/min and 2.79 ± 0.57 μL/min in the young and the aged, respectively. CSF flow rates were 3.74 ± 1.93 μL/min and 3.19 ± 0.70 μL/min at the 3rd ventricle and 2.89 ± 0.21 μL/min and 2.70 ± 1.29 μL/min at the 4th ventricle (Figure 4A- 4C). The total intracranial CSF volume was 204 ± 97 μL in the young and 275 ± 35 μL in the aged cohort (Figure 4D). None of these values differed significantly between the young vs. aged cohorts.

### Brain volume

**Figure 5:**
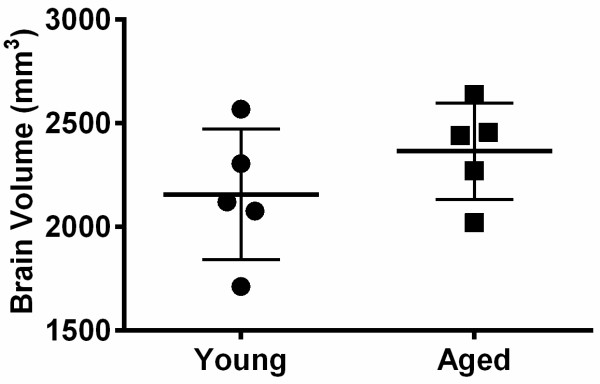
**Total brain volume calculated from baseline non-contrast images.** Young (2–5 months); Aged (16 months). Mean ± SD.

Brain volume was 2157 ± 314 mm^3^ and 2366 ± 233 mm^3^ in the young and aged rats, respectively (Figure 5). CSF volumes (and as percentage of brain volume) were 204 ± 97 μL (8.8 ± 4.3%) in the young and 275 ± 35 μL (10.8 ± 1.9%) in the aged animals. These values were not significantly different.

## Discussion

This study used young and aged SHRs to demonstrate a new technique for the measurement of CSF flow, volume and drainage *in vivo*. The results of this study suggest that sagittal sinus arachnoid granulations are not the primary route of CSF drainage and that CSF is primarily absorbed via the spinal and olfactory routes. The time taken for contrast-enhanced CSF to reach each drainage route did not differ between the young and aged animals. Our novel CT method also allowed for measurement of CSF flow and volume and found that there was little difference between young and aged animals, despite a higher ICP in the aged animals. This method provides an alternative avenue for the investigation of the pathophysiological perturbations occurring in disorders of CSF regulation and abnormal intracranial pressure.

There is increasing recognition of the importance of altered CSF and brain interstitial fluid dynamics with age, not only in conditions such as normal pressure hydrocephalus but also in dementias such as Alzheimer’s disease [44,45]. Despite this, most CSF-related research occurs in young animals. In the current study we investigated CSF dynamics in an aged population of SHRs, since this strain is known to develop hypertension, ventriculomegaly and cerebral volume loss with age [36,37]. We found little difference in the absolute CSF flow rates or total brain volume between young (2–5 months) and aged (16 months) rats. These data are similar to findings in a previous study of normotensive rats (without ventriculomegaly) aged from 3–30 months. Chiu et al. found that peak CSF production occurs at 10 months before steadily decreasing over time to almost baseline values [43]. Furthermore, the values they reported in 12–20 month rats ranged from 2.66-2.84 μL/min, which were comparable to our reported values in 16 month aged rats and to previously reported values in 3–4 months old rats in other studies [46,47]. It is also interesting to note that the CSF flow rates, when measured at different anatomical locations within the CSF ventricular system, did not vary greatly. The lack of a significant difference in *in vivo* CSF volumes between young and aged SHRs is inconsistent with previous *in vitro* studies, and most likely due to a lack of statistical power to detect a difference in this variable. This was contributed to by small animal numbers and in particular by the significant between-animal variability in the younger cohort. The point estimate of a 26% difference is in keeping with previous published data [36,37]. Additionally, it may be that changes would be more apparent in ‘elderly’ (≈2 years) animals, than in at 16 months of age. This age was chosen to avoid the tumours and mortality that increase beyond 18 months in this strain. Our data also demonstrated a significantly higher intracranial pressure (ICP) and cerebral perfusion pressure (CPP) in the aged rats when compared to the young. This is consistent with findings in humans [48,49] and in other rat strains [50]. Additionally, the greater variability in CPP in the young animals may have contributed to the greater variance in CSF data, i.e. there was a greater percentage of variance (relative to the mean) in mean arterial pressure, and in ICP, in the young vs. aged. It is plausible that greater variance in CPP resulted in greater variance in CSF flow and drainage parameters, as changes in CPP may affect CSF production and drainage.

Although the ICP was higher in aged animals, we could not see any contrast adjacent to or filling the sagittal sinus to indicate drainage of the CSF via arachnoid granulations, even at the later time points. There was, however, clear evidence of passage of contrast into the spinal canal, olfactory cavity, along the ophthalmic nerves and into cervical lymph nodes. Our findings support recent studies that the primary route of CSF drainage in most mammalian species is via perineural sheaths. In anatomical studies using a coloured tracer and anatomical dissection, CSF drainage into nasal lymphatics via the olfactory nerves and cribriform plate has been demonstrated in sheep, pigs, rabbits, rats, mice and monkeys [16,17,20-25]. Additionally presence of this route has been shown in human cadavers [20]. Many studies have also demonstrated spinal lymphatic CSF drainage using tracers in several mammalian species including humans [22,23,26,35,51]. It was calculated that the rate of spinal CSF absorption was between 38–76% of CSF production in healthy individuals (higher during activity) [35]. At least in the sheep, the venous sinus arachnoid granulation pathway of CSF absorption appears to be a secondary pathway only recruited at high intracranial pressures, for example after a neurological injury [17,28].

The calculation of CSF volume is technically challenging and many techniques have been tried, with varying success. Values obtained in this study correspond well to some published data in rats using quite different techniques, including volumes of 233–240 μL in young SHRs using the ventriculo-cisternal dilution method [46,47] and volumes from 275–441 μL in Fischer 344/BN rats using magnetic resonance imaging [43]. However, some published studies report much higher volumes. Lai et al. (1983) reported a mean CSF volume of 580 μL in rats using the formula ‘CSF volume = CSF formation rate/CSF turnover rate’ [52]. They assumed that the CSF turnover rate was constant amongst species [53], and used the human CSF turnover rate of 0.38% per minute (obtained using ventriculo-lumbar perfusion method from 12 children with subacute sclerosing panencephalitis and pontine glioma) [54], to calculate CSF volume. We are not sure that this assumption is well justified. However, despite these potential limitations, the rat CSF volume and turnover rate from that paper appear to have become the accepted values in reviews of the field, perhaps due to the paucity of other available data [55-57].

Our novel CT method provides several possible advantages, and some limitations, when compared with other techniques such as the ventriculo-cisternal dilution and post-mortem dye-tracer methods. First among these is that it does not require an intracisternal draining catheter, with potential resultant effects on ICP and possibly on CSF production, if homeostatic mechanisms are evoked. Secondly, the ability to image the entire CSF system simultaneously and sequentially gives a more complete understanding of the dynamics of CSF flow and drainage. We were able to monitor the major physiological parameters thought to influence CSF production rate, that is, ICP [58], blood pressure [59], and temperature [60]. Additionally, flow rates, whole brain volume, and CSF volume calculations were obtained from the same study. Some unavoidable limitations also exist with the CT method, and particular points of the analysis require great care. In particular, partial volume averaging effects are a known potential problem when measuring values from a very small structure such as the cerebral aqueduct. Great care must be taken to identify the midpoint of the region of contrast enhancement for placement of a small region-of-interest. An additional limitation is that although we could observe and quantify the time taken for contrast-enhanced CSF to reach the drainage pathways *in vivo*, we have been unable to reliably quantify the volume of CSF draining via these routes.

## Conclusions

We have provided *in vivo* data using CT imaging of CSF distribution over time, which indicates that the primary route of CSF drainage in young and aged rats is via the spinal and olfactory lymphatics, and that drainage into the sagittal sinus arachnoid granulations plays at most a minor role. The CT technique we developed provides an alternative to ventriculo-cisternal dilution methods for measurement of CSF flow through the cerebral aqueduct, the widely accepted surrogate measure for CSF production. It avoids the need to puncture the cisterna magna and permits visualisation and timing of CSF distribution and drainage, quantification of CSF flow elsewhere within the ventricular system and measurement of total brain and intracranial CSF volumes. Interestingly, this study of young and aged SHRs suggest that CSF production rates and volumes are quite similar, and do not change dramatically with age. The information gathered using our novel contrast-enhanced CT method may provide much needed insight into the CSF dynamics and drainage involved in many neurological diseases.

## Abbreviations

CSF: Cerebrospinal fluid; CT: Computed tomography; HU: Hounsfield Units; ICP: Intracranial pressure; MIP: Maximum Intensity Projection; ROI: Region of interest; SHR: Spontaneously hypertensive rat.

## Competing interests

The authors declare that they have no competing interests.

## Authors’ contributions

LM and DM carried out the surgical and computed tomography components of the study, analysed and interpreted the data, performed statistical analysis and drafted the manuscript. QY designed MiStar software decay module specifically written for this project. DB and NS participated in the design of the study and helped draft the manuscript. DM, NS, LM, MP and CL, conceived the study, and participated in its design and coordination. All authors read and approved the final manuscript.

## Authors’ information

LM- B. Biomed Sci. (Hons), PhD Candidate (Human Physiology).

QY- PhD (Physics).

MP- PhD, FRACP (Neurology).

CL- MBBS, B Med Sci (Hons), FRACP (Neurology).

DB- B. Biomed Sci. (Hons), PhD Candidate (Human Physiology).

NS- MBBS, PhD, FRACP (Neurology).

DM- PhD (Human Physiology).

## Supplementary Material

Additional file 1 **Contrast-enhanced cerebrospinal fluid flow through the cranium over 60 minutes.** Radio-opaque contrast (20 μl) was simultaneously injected into each lateral ventricle at 2 μl/min for 10 min while plain CT images (0.6 mm slice thickness) were taken over 60 minutes. File format: mov. Click here for file

Additional file 2 **3D render reconstruction of cerebrospinal fluid system of the rat.** The computed tomography images of one rat were loaded in MiStar software and subtracted from the baseline non-contrast image. A Maximum Instensity Projection was generation and loaded into the MiStar fusion 3D render module. File format: mov. Click here for file

## References

[B1] Wagshul ME McAllister JP Rashid S Li J Egnor MR Walker ML Yu M Smith SD Zhang G Chen JJ Benveniste H Ventricular dilation and elevated aqueductal pulsations in a new experimental model of communicating hydrocephalus Exp Neurol 2009 218 33 40 10.1016/j.expneurol.2009.03.034 19348801

[B2] Bateman GA The pathophysiology of idiopathic normal pressure hydrocephalus: cerebral ischemia or altered venous hemodynamics? AJNR Am J Neuroradiol 2008 29 198 203 10.3174/ajnr.A0739 17925373 PMC8119093

[B3] Preuss M Hoffmann KT Reiss-Zimmermann M Hirsch W Merkenschlager A Meixensberger J Dengl M Updated physiology and pathophysiology of CSF circulation–the pulsatile vector theory Childs Nerv Syst 2013 29 1811 1825 10.1007/s00381-013-2219-0 23832074

[B4] Killer HE Jaggi GP Flammer J Miller NR Huber AR Mironov A Cerebrospinal fluid dynamics between the intracranial and the subarachnoid space of the optic nerve. Is it always bidirectional? Brain 2007 130 514 520 10.1093/brain/awl324 17114796

[B5] Shapira Y Artru AA Lam AM Changes in the rate of formation and resistance to reabsorption of cerebrospinal fluid during deliberate hypotension induced with adenosine or hemorrhage Anesthesiology 1992 76 432 439 10.1097/00000542-199203000-00017 1539856

[B6] Paradot G Baledent O Gondry-Jouet C Meyer ME Le Gars D [Cerebrospinal fluid flow imaging in the meningeal hemorrhage] Neurochirurgie 2006 52 323 329 10.1016/S0028-3770(06)71226-7 17088712

[B7] Brinker T Seifert V Stolke D Acute changes in the dynamics of the cerebrospinal fluid system during experimental subarachnoid hemorrhage Neurosurgery 1990 27 369 372 10.1227/00006123-199009000-00005 2234329

[B8] Schwab S Schellinger P Aschoff A Albert F Spranger M Hacke W [Epidural cerebrospinal fluid pressure measurement and therapy of intracranial hypertension in “malignant” middle cerebral artery infarct] Nervenarzt 1996 67 659 666 10.1007/s001150050038 8805111

[B9] Minnerup J Wersching H Ringelstein EB Heindel W Niederstadt T Schilling M Schabitz WR Kemmling A Prediction of malignant middle cerebral artery infarction using computed tomography-based intracranial volume reserve measurements Stroke 2011 42 3403 3409 10.1161/STROKEAHA.111.619734 21903965

[B10] Johanson C Stopa E Baird A Sharma H Traumatic brain injury and recovery mechanisms: peptide modulation of periventricular neurogenic regions by the choroid plexus-CSF nexus J Neural Transm 2011 118 115 133 10.1007/s00702-010-0498-0 20936524 PMC3026679

[B11] Schmid Daners M Knobloch V Soellinger M Boesiger P Seifert B Guzzella L Kurtcuoglu V Age-specific characteristics and coupling of cerebral arterial inflow and cerebrospinal fluid dynamics PLoS One 2012 7 e37502 10.1371/journal.pone.0037502 22666360 PMC3364266

[B12] Stoquart-ElSankari S Baledent O Gondry-Jouet C Makki M Godefroy O Meyer ME Aging effects on cerebral blood and cerebrospinal fluid flows J Cereb Blood Flow Metab 2007 27 1563 1572 10.1038/sj.jcbfm.9600462 17311079

[B13] Tripathi R Tracing the bulk outflow route of cerebrospinal fluid by transmission and scanning electron microscopy Brain Res 1974 80 503 506 10.1016/0006-8993(74)91033-6 4214009

[B14] Tripathi BJ Tripathi RC Vacuolar transcellular channels as a drainage pathway for cerebrospinal fluid J Physiol 1974 239 195 206 4369428 10.1113/jphysiol.1974.sp010563 PMC1330945

[B15] Welch K Friedman V The cerebrospinal fluid valves Brain 1960 83 454 469 10.1093/brain/83.3.454 13784191

[B16] Cserr HF Harling-Berg CJ Knopf PM Drainage of brain extracellular fluid into blood and deep cervical lymph and its immunological significance Brain Pathol 1992 2 269 276 10.1111/j.1750-3639.1992.tb00703.x 1341962

[B17] Zakharov A Papaiconomou C Koh L Djenic J Bozanovic-Sosic R Johnston M Integrating the roles of extracranial lymphatics and intracranial veins in cerebrospinal fluid absorption in sheep Microvasc Res 2004 67 96 104 10.1016/j.mvr.2003.08.004 14709407

[B18] Kida S Pantazis A Weller RO CSF drains directly from the subarachnoid space into nasal lymphatics in the rat. Anatomy, histology and immunological significance Neuropathol Appl Neurobiol 1993 19 480 488 10.1111/j.1365-2990.1993.tb00476.x 7510047

[B19] Carare RO Hawkes CA Weller RO Afferent and efferent immunological pathways of the brain. Anatomy, function and failure Brain Behav Immun 2014 36 9 14 24145049 10.1016/j.bbi.2013.10.012

[B20] Johnston M Zakharov A Papaiconomou C Salmasi G Armstrong D Evidence of connections between cerebrospinal fluid and nasal lymphatic vessels in humans, non-human primates and other mammalian species Cerebrospinal Fluid Res 2004 1 2 10.1186/1743-8454-1-2 15679948 PMC546409

[B21] Nagra G Koh L Zakharov A Armstrong D Johnston M Quantification of cerebrospinal fluid transport across the cribriform plate into lymphatics in rats Am J Physiol Regul Integr Comp Physiol 2006 291 R1383 R1389 10.1152/ajpregu.00235.2006 16793937

[B22] Johnston M Zakharov A Koh L Armstrong D Subarachnoid injection of Microfil reveals connections between cerebrospinal fluid and nasal lymphatics in the non-human primate Neuropathol Appl Neurobiol 2005 31 632 640 10.1111/j.1365-2990.2005.00679.x 16281912

[B23] Boulton M Young A Hay J Armstrong D Flessner M Schwartz M Johnston M Drainage of CSF through lymphatic pathways and arachnoid villi in sheep: measurement of 125I-albumin clearance Neuropathol Appl Neurobiol 1996 22 325 333 10.1111/j.1365-2990.1996.tb01111.x 8875467

[B24] Boulton M Flessner M Armstrong D Mohamed R Hay J Johnston M Contribution of extracranial lymphatics and arachnoid villi to the clearance of a CSF tracer in the rat Am J Physiol 1999 276 R818 R823 10070143 10.1152/ajpregu.1999.276.3.R818

[B25] Boulton M Flessner M Armstrong D Hay J Johnston M Lymphatic drainage of the CNS: effects of lymphatic diversion/ligation on CSF protein transport to plasma Am J Physiol 1997 272 R1613 R1619 9176355 10.1152/ajpregu.1997.272.5.R1613

[B26] Silver I Li B Szalai J Johnston M Relationship between intracranial pressure and cervical lymphatic pressure and flow rates in sheep Am J Physiol 1999 277 R1712 R1717 10600918 10.1152/ajpregu.1999.277.6.R1712

[B27] Mollanji R Bozanovic-Sosic R Zakharov A Makarian L Johnston MG Blocking cerebrospinal fluid absorption through the cribriform plate increases resting intracranial pressure Am J Physiol Regul Integr Comp Physiol 2002 282 R1593 R1599 12010739 10.1152/ajpregu.00695.2001

[B28] Papaiconomou C Zakharov A Azizi N Djenic J Johnston M Reassessment of the pathways responsible for cerebrospinal fluid absorption in the neonate Childs Nerv Syst 2004 20 29 36 10.1007/s00381-003-0840-z 14605840

[B29] Welch K Pollay M The spinal arachnoid villi of the monkeys Cercopithecus aethiops sabaeus and Macaca irus Anat Rec 1963 145 43 48 10.1002/ar.1091450107 13999821

[B30] Gomez DG Chambers AA Di Benedetto AT Potts DG The spinal cerebrospinal fluid absorptive pathways Neuroradiology 1974 8 61 66 10.1007/BF00345037 4140480

[B31] Kido DK Gomez DG Pavese AM Jr Potts DG Human spinal arachnoid villi and granulations Neuroradiology 1976 11 221 228 10.1007/BF00328377 980235

[B32] Tubbs RS Hansasuta A Stetler W Kelly DR Blevins D Humphrey R Chua GD Shoja MM Loukas M Oakes WJ Human spinal arachnoid villi revisited: immunohistological study and review of the literature J Neurosurg Spine 2007 7 328 331 10.3171/SPI-07/09/328 17877268

[B33] Marmarou A Shulman K LaMorgese J Compartmental analysis of compliance and outflow resistance of the cerebrospinal fluid system J Neurosurg 1975 43 523 534 10.3171/jns.1975.43.5.0523 1181384

[B34] Bozanovic-Sosic R Mollanji R Johnston MG Spinal and cranial contributions to total cerebrospinal fluid transport Am J Physiol Regul Integr Comp Physiol 2001 281 R909 R916 11507008 10.1152/ajpregu.2001.281.3.R909

[B35] Edsbagge M Tisell M Jacobsson L Wikkelso C Spinal CSF absorption in healthy individuals Am J Physiol Regul Integr Comp Physiol 2004 287 R1450 R1455 10.1152/ajpregu.00215.2004 15308484

[B36] Ritter S Dinh TT Progressive postnatal dilation of brain ventricles in spontaneously hypertensive rats Brain Res 1986 370 327 332 10.1016/0006-8993(86)90488-9 3708330

[B37] Ritter S Dinh TT Stone S Ross N Cerebroventricular dilation in spontaneously hypertensive rats (SHRs) is not attenuated by reduction of blood pressure Brain Res 1988 450 354 359 10.1016/0006-8993(88)91574-0 3042092

[B38] Murtha LA McLeod DD McCann SK Pepperall D Chung S Levi CR Calford MB Spratt NJ Short-duration hypothermia after ischemic stroke prevents delayed intracranial pressure rise Int J Stroke 2013 doi:10.1111/ijs.12181 10.1111/ijs.12181 24025084

[B39] Artru AA Isoflurane does not increase the rate of CSF production in the dog Anesthesiology 1984 60 193 197 10.1097/00000542-198403000-00004 6696252

[B40] Murtha L McLeod D Spratt N Epidural intracranial pressure measurement in rats using a fiber-optic pressure transducer J Vis Exp 2012 62 e3689 doi:10,3791/3689 10.3791/3689 PMC3466637 22565931

[B41] McLeod D Parsons M Hood R Hiles B Allen J McCann S Murtha L Calford M Levi C Spratt N Perfusion computed tomography thresholds defining ischaemic penumbra and infarct core: studies in a rat stroke model Int J Stroke 2013 doi: 10.1111/ijs.12147 10.1111/ijs.12147 24138577

[B42] McLeod DD Parsons MW Levi CR Beautement S Buxton D Roworth B Spratt NJ Establishing a rodent stroke perfusion computed tomography model Int J Stroke 2011 6 284 289 10.1111/j.1747-4949.2010.00564.x 21609409

[B43] Chiu C Miller MC Caralopoulos IN Worden MS Brinker T Gordon ZN Johanson CE Silverberg GD Temporal course of cerebrospinal fluid dynamics and amyloid accumulation in the aging rat brain from three to thirty months Fluids Barriers CNS 2012 9 3 10.1186/2045-8118-9-3 22269091 PMC3274479

[B44] Xie L Kang H Xu Q Chen MJ Liao Y Thiyagarajan M O’Donnell J Christensen DJ Nicholson C Iliff JJ Takano T Deane R Nedergaard M Sleep drives metabolite clearance from the adult brain Science 2013 342 373 377 10.1126/science.1241224 24136970 PMC3880190

[B45] Sykova E Vorisek I Antonova T Mazel T Meyer-Luehmann M Jucker M Hajek M Ort M Bures J Changes in extracellular space size and geometry in APP23 transgenic mice: a model of Alzheimer’s disease Proc Natl Acad Sci U S A 2005 102 479 484 10.1073/pnas.0408235102 15630088 PMC544312

[B46] Al-Sarraf H Philip L Effect of hypertension on the integrity of blood brain and blood CSF barriers, cerebral blood flow and CSF secretion in the rat Brain Res 2003 975 179 188 10.1016/S0006-8993(03)02632-5 12763606

[B47] Al-Sarraf H Philip L Increased brain uptake and CSF clearance of 14C-glutamate in spontaneously hypertensive rats Brain Res 2003 994 181 187 10.1016/j.brainres.2003.09.034 14642643

[B48] Dunn LT Raised intracranial pressure J Neurol Neurosurg Psychiatry 2002 73 Suppl 1 i23 i27 12185258 10.1136/jnnp.73.suppl_1.i23 PMC1765599

[B49] Rangel Castilla L Gopinath S Robertson CS Management of intracranial hypertension Neurol Clin 2008 26 521 541 x 10.1016/j.ncl.2008.02.003 18514825 PMC2452989

[B50] Hawkins BE Cowart JC Parsley MA Capra BA Eidson KA Hellmich HL Dewitt DS Prough DS Effects of trauma, hemorrhage and resuscitation in aged rats Brain Res 2013 1496 28 35 23274538 10.1016/j.brainres.2012.12.027 PMC3569488

[B51] Ghersi-Egea JF Finnegan W Chen JL Fenstermacher JD Rapid distribution of intraventricularly administered sucrose into cerebrospinal fluid cisterns via subarachnoid velae in rat Neuroscience 1996 75 1271 1288 10.1016/0306-4522(96)00281-3 8938759

[B52] Lai YL Smith PM Lamm WJ Hildebrandt J Sampling and analysis of cerebrospinal fluid for chronic studies in awake rats J Appl Physiol 1983 54 1754 1757 6409862 10.1152/jappl.1983.54.6.1754

[B53] Cserr HF Physiology of the choroid plexus Physiol Rev 1971 51 273 311 4930496 10.1152/physrev.1971.51.2.273

[B54] Cutler RW Page L Galicich J Watters GV Formation and absorption of cerebrospinal fluid in man Brain 1968 91 707 720 10.1093/brain/91.4.707 5304069

[B55] Johanson CE Duncan JA 3rd Klinge PM Brinker T Stopa EG Silverberg GD Multiplicity of cerebrospinal fluid functions: New challenges in health and disease Cerebrospinal Fluid Res 2008 5 10 10.1186/1743-8454-5-10 18479516 PMC2412840

[B56] Preston JE Ageing choroid plexus-cerebrospinal fluid system Microsc Res Tech 2001 52 31 37 10.1002/1097-0029(20010101)52:1<31::AID-JEMT5>3.0.CO;2-T 11135446

[B57] Redzic ZB Preston JE Duncan JA Chodobski A Szmydynger-Chodobska J The choroid plexus-cerebrospinal fluid system: from development to aging Curr Top Dev Biol 2005 71 1 52 16344101 10.1016/S0070-2153(05)71001-2

[B58] Hochwald GM Sahar A Effect of spinal fluid pressure on cerebrospinal fluid formation Exp Neurol 1971 32 30 40 10.1016/0014-4886(71)90162-2 5095607

[B59] Carey ME Vela AR Effect of systemic arterial hypotension on the rate of cerebrospinal fluid formation in dogs J Neurosurg 1974 41 350 355 10.3171/jns.1974.41.3.0350 4413096

[B60] Snodgrass SR Lorenzo AV Temperature and cerebrospinal fluid production rate Am J Physiol 1972 222 1524 1527 5030212 10.1152/ajplegacy.1972.222.6.1524

[B61] Paxinos G Watson C The rat brain in stereotaxic coordinates 1998 4 San Diego: Academic Press 10.1016/0165-0270(80)90021-7 6110810

